# Natural Killer Cells: Friend or Foe in Metabolic Diseases?

**DOI:** 10.3389/fimmu.2021.614429

**Published:** 2021-02-24

**Authors:** Yi Li, Fangjie Wang, Saber Imani, Ling Tao, Youcai Deng, Yue Cai

**Affiliations:** ^1^ Department of Cardiology, Xijing Hospital, Fourth Military Medical University, Xi’an, China; ^2^ Student Brigade, Preclinical School of Medicine, The Fourth Military Medical University, Xi’an, China; ^3^ Department of Anesthesiology and Perioperative Medicine, Xijing Hospital, The Fourth Military Medical University, Xi’an, China; ^4^ State Key Laboratory of Trauma, Burns and Combined Injury, Department of Wound Infection and Drug, Daping Hospital, Army Medical University (Third Military Medical University), Chongqing, China; ^5^ Department of Oncology, The Affiliated Hospital of Southwest Medical University, Luzhou, China; ^6^ Institute of Materia Medica, College of Pharmacy, Army Medical University (Third Military Medical University), Chongqing, China

**Keywords:** natural killer cell, metabolic syndrome, obesity, insulin resistance, type 2 diabetes mellitus, nonalcoholic fatty liver disease, atherosclerosis

## Abstract

The worldwide epidemic of metabolic diseases, especially obesity and other diseases caused by it, has shown a dramatic increase in incidence. A great deal of attention has been focused on the underlying mechanisms of these pathological processes and potential strategies to solve these problems. Chronic inflammation initiated by abdominal adipose tissues and immune cell activation in obesity is the major cause of the consequent development of complications. In addition to adipocytes, macrophages and monocytes, natural killer (NK) cells have been verified to be vital components involved in shaping the inflammatory microenvironment, thereby leading to various obesity-related metabolic diseases. Here, we provide an overview of the roles of NK cells and the interactions of these cells with other immune and nonimmune cells in the pathological processes of metabolic diseases. Finally, we also discuss potential therapeutic strategies targeting NK cells to treat metabolic diseases.

## Introduction

The increase in high calorie-low fiber fast food consumption and the decrease in physical activity have led to a rapidly increasing incidence of metabolic diseases in most countries throughout the world (1, 2). Obesity, which is characterized by excessive fat accumulation and diagnosed at a body mass index (BMI) ≥30 kg/m^2^, is considered the major risk factor for the development of several metabolic diseases, such as insulin resistance, diabetes mellitus (DM), nonalcoholic fatty liver disease (NAFLD), atherosclerosis, and hypertension (3–5). Visceral adipose tissue (VAT) is a main part of white adipose tissue and acts as a site of adipocyte hyperplasia and hypertrophy during obesity. VAT is involved in a sequence of detrimental processes in obesity that leads to increased adipocyte hypoxia, fatty acid flux dysregulation, increased chemokine secretion and extracellular matrix protein expression, adipocyte cell death, and proinflammatory cell recruitment (6). These proinflammatory immune cells can release abundant inflammatory cytokines and induce serine phosphorylation of insulin receptor substrate-1, leading to local and systemic insulin resistance and dysregulation of glucose and fatty acid metabolism (6).

Among all of the factors mentioned above, the innate and adaptive immune systems have received much more attention recently and are widely accepted as forces that participate in the development of metabolic diseases. It is well established that the initiation and progression of obesity-related metabolic diseases are dependent on low-grade, chronic systemic inflammation induced by various cellular stress responses in adipose tissues and immune cell activation (7). During obesity, macrophages, lymphocytes, dendritic cells (DCs) and many other immune cells produce inflammatory mediators that interact with each other to contribute to the disturbance (6). To date, accumulating evidence has revealed that natural killer (NK) cells play a significant role in this pathological condition (8–11). In this review, we will summarize the current knowledge on the alterations in NK cell effector functions, possible mechanisms and the interactions of NK cells with regional adjacent tissue cells in metabolic diseases.

## A Brief Introduction to NK Cells

NK cells are identified as one group of innate lymphoid cells (ILCs); they are generated from common innate lymphoid progenitors in the bone marrow and then migrate to various lymphoid or nonlymphoid tissues, including the thymus, spleen, lymph nodes, lungs, liver, kidneys and VAT, where they become resident (12, 13). In the blood, NK cells represent 1–6% of all leukocytes; however, they are reported to account for approximately 13% of VAT leukocytes (14).

NK cells mediate cellular cytotoxicity to recognize and kill infected or cancerous cells rapidly and regulate other cells under physiological and pathological conditions through receptor-ligand interactions or cytokine/chemokine production (15). NK cell functions are mainly regulated by the expression of germline-encoded activating and inhibitory receptors on the cell surface. The most dominant inhibitory receptors are killer cell immunoglobulin-like receptors (KIRs) and killer cell lectin-like receptors (KLRs), which recognize classical and nonclassical major histocompatibility complex (MHC) class I molecules on the surface of target cells (16). In mice, the function that is performed by killer immunoglobulin-like receptors (KIRs) in human NK cells is assigned to the Ly49 receptors (17). While some activating receptors, such as NCRs (NKp46, NKp30, NKp44, etc.), NKG2D, and DNAM-1, are involved in triggering NK cell effector functions (16). NK cells also produce many cytokines and chemokines, such as tumor necrosis factor (TNF)-α, interferon (IFN)-γ, granulocyte macrophage colony-stimulating factor (GM-CSF), interleukin (IL)-5, IL-10, IL-13, and CCL3-5, in response to stimulation, leading to the activation of DCs, macrophages or adjacent cells in situ (18). In turn, cytokines, including IFN-α/β, IL-12, IL-18, and IL-15 from activated DCs, macrophages or infected tissues, can facilitate NK cell cytotoxicity, induce IFN-γ production and promote cell proliferation (19, 20). Thus, through a feedback loop, NK cells participate in amplifying inflammatory responses and shaping an inflammatory microenvironment. As inflammation plays a detrimental role in obesity and related metabolic diseases, the functions of NK cells under these conditions are worthy of deeper investigation.

## NK Cells in Obesity

In view of the fact that not all obese individuals develop obesity-associated metabolic diseases, these patients are termed “metabolically healthy obese”, and it has been proposed that only chronic systematic inflammation induces the initiation and progression of obesity-associated metabolic diseases (21, 22). Adipose tissue depots are made up of various components, including adipocytes, preadipocytes, endothelial cells, and several immune cell types that shape the immunological microenvironment (23). In humans, several studies have revealed an obvious reduction in the NK cell number in the peripheral blood in obese adults or adolescents (24, 25). Moreover, murine models of obesity demonstrate NK cell accumulation in adipose tissue (14, 26).

**Figure 1:**
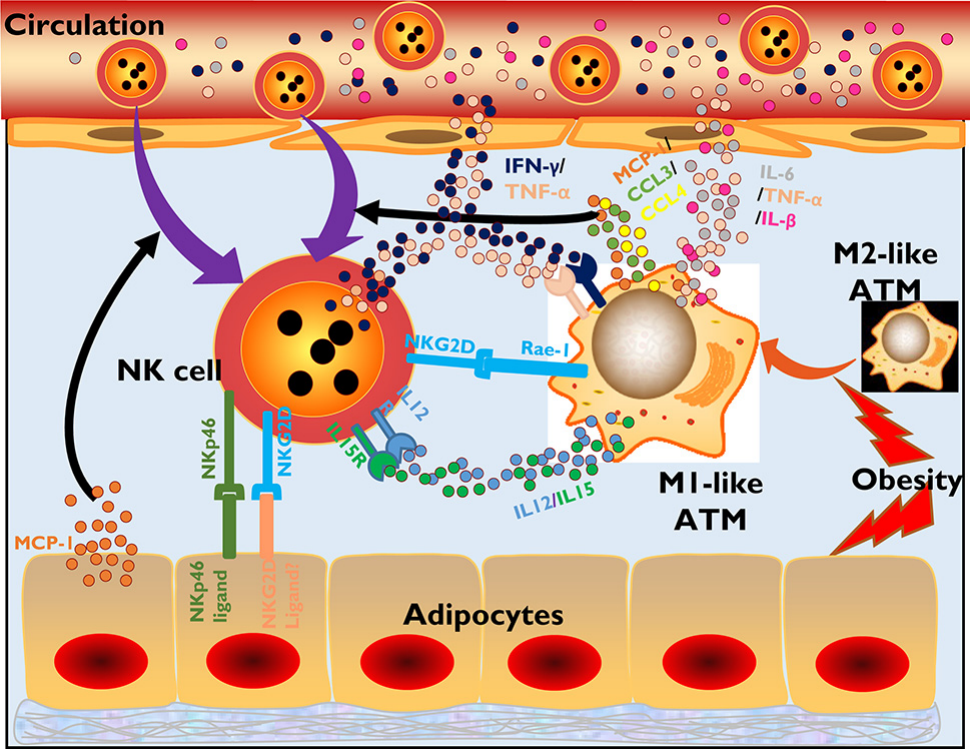
NK cells participate in the inflammatory initiation of obesity-related diseases. In obesity, adipocytes tend to secrete MCP-1, recruiting more NK cells and monocyte/macrophages to infiltrate adipose tissues. Adipocytes also express ligands of NKp46 and NKG2D, which have not been specifically defined, to activate NK cells through signaling of surface-expressed activating receptors. Moreover, obesity promotes ATM polarization from an M2-like phenotype to an M1-like phenotype, which is a proinflammatory phenotype. M1-like ATMs secrete the chemokines MCP-1, CCL3, and CCL4 or cytokines IL-12 and IL-15, which also recruit NK cells and promote NK cell activation and proliferation. ATMs also express the NKG2D-specific ligand Rae-1. Activated NK cells in adipose tissue secrete inflammatory cytokines, such as IFN-γ and TNF-α, which also promote ATM polarization and activation. Finally, the large amounts of inflammatory cytokines produced by activated NK cells and ATMs in adipose tissue are released into the circulation, causing systemic inflammation and various metabolic diseases.

Adipocytes play a significant role in NK cell activation. When obesity occurs, the excessive nutrient load causes obvious cellular stresses, including hypoxia, mechanical stress, ER stress and lipid accumulation, in adipocytes (9). Cellular stresses promote preferential secretion of MCP-1 (C-C motif chemokine ligand 2, also called as CCL-2) by adipocytes, a potent chemoattractant for monocytes and macrophages, which are important in initiating adipose tissue inflammation (27). MCP-1 drives recruitment of peripheral NK cells into the adipose tissue (28). These NK cells are reported to express higher levels of activation receptors, including NKG2D, CD158 and NKp46, when compared to peripheral blood NK cells. Adipose tissue NK cells in obese subjects also showed an increased expression of NKG2D than that in lean subjects (29). In addition, obesity also drives the upregulation of an unidentified ligand of the NK cell activating receptor NKp46 on adipocytes, which promotes NK cell proliferation and activation (14) (**Figure 1**).

Under lean conditions, macrophages are the most dominant immune cells in adipose tissue, and most of them maintain an M2-like phenotype, which regulates anti-inflammatory factors. However, obesity introduces a phenotypic transition in macrophages from an M2-like phenotype to an M1-like phenotype, a proinflammatory transition (30). Under these conditions, adipose tissue-resident macrophages (ATMs) secrete more chemokines, including MCP-1, CCL3 and CCL4, recruiting NK cells into the adipose tissue from the circulation (26). Moreover, these macrophages tend to generate more IL-15 or IL-12, promoting NK cell activation and proliferation in situ (26). In response to obesity, ATMs also upregulate the expression of Rae-1 (also known as RAET-1 in humans), a kind of NKG2D ligand that potentially triggers NK cell activation (31) (**Figure 1**).

The activated NK cells in adipose tissue in the context of obesity will in turn participate in shaping the inflammatory microenvironment, especially the activation status of ATMs. In fact, NK cells are reported to account for ~43% of the IFN-γ-producing population in adipose tissue, which promotes and maintains the M1 polarization of macrophages and inflammatory gene expression in adipocytes (14, 26). Depletion of NK cells directly reduces the number of infiltrated ATMs and their expression of inflammatory genes, including *Tnf* and *Il1b*, while promoting anti-inflammatory *IL-10* and *Arg1* expression (26). In addition, the absence of NK cells in obesity will also reduce adipocyte expression of MCP-1, which is a chemokine that attracts monocytes into adipose tissue (26). Moreover, the frequency of NKG2D^+^ NK cells in the peripheral blood of patients with type 2 diabetes mellitus (T2DM) is significantly higher than that in the peripheral blood of controls and has been demonstrated to be correlated with BMI values (32). However, the effect of this subtype has not yet been reported, and unlike in NAFLD and atherosclerosis, which are discussed below, obesity-induced inflammation and insulin resistance development seem to exhibit an absence of the NKG2D-NKG2D ligand interaction in ATMs (33). However, in contrast, the normal cytotoxic potential of NK cells seems to be impaired, with lower expression of cytolytic granules, including granzymes and perforin, or a damaged ability to kill cancer cells *in vitro* (24).

Intriguingly, it has been reported that the influence of obesity on NK cells, as well as the interaction between NK and ATMs mentioned above, occurs only in epididymal, not subcutaneous, adipose tissue of C57BL/6 mice (26). Using anti-asialo-glycolipid asialoganglioside M1 (GM1) antibody, anti-NK1.1 antibody (PK-136), or E4bp4 knockout mice to deplete NK cells could improve high-fat diet-induced insulin resistance in parallel with decreases in both ATM numbers and adipose tissue inflammation (26). It is not clear why NK cells have selectivity for the location of adipose tissue, but the distinct components and microenvironment of the adipose tissue may matter. Moreover, dysregulated NK cells from obese rats were shown to be ameliorated by being transferred into normal-weight rats (34). The weight loss induced by surgery or exercise training can also modify the production of cytokines and cytotoxicity of NK cells (35, 36). This phenomenon may be explained by the decrease of leptin, increase of adiponectin or reduced lipid accumulation in NK after weight loss in obese individuals (28, 37–39). Previous studies have revealed that *in vitro* 100 ng/ml leptin treatment, which indicates obesity *in vivo*, led to increased expression of IFN-γ, perforin, granzyme B, and CD69, together with increased direct cytotoxicity by human NK cell lines, including NK92 and YT cells (40, 41). Short-term treatment (24 h) of leptin on human primary NK cells treated with leptin *in vitro* also showed increased direct cytotoxicity and IFN-γ; however, long-term leptin treatment showed the opposite results (42). In addition, adiponectin negatively regulates NK cell IFN-γ production and cytotoxicity (43, 44). Xavier Michelet et al. reveals that obesity results in peroxisome proliferator-activated receptor (PPAR) signal-driven lipid accumulation in NK cells, causing blunted anti-tumor responses, while blocking lipid transportation into NK mitochondria could significantly reverse the cytotoxic paralysis (39). All of this evidence suggests that in the early stage of obesity, increased leptin levels and/or decreased adiponectin levels enhance NK cell activation and contribute to developing or exacerbating the inflammatory response in adipose tissue. However, when obesity is persistent, leptin or intra-cellular lipid accumulation leads to the immune paralysis of NK cells, which might be an explanation for the higher cancer incidence in individuals with obesity (39, 45).

## NK Cells in Insulin Resistance and T2DM

As one of the well-known complications of obesity, insulin resistance can further lead to T2DM. Obesity-induced inflammation is generally considered one of the major causes of the development of insulin resistance and T2DM (46). Proinflammatory cytokines, especially IFN-γ, TNF-α and IL-1β, can impair insulin signaling in peripheral tissues or induce β-cell dysfunction and subsequent insulin deficiency. In particular, IL-1β is generated from saturated fatty acids through the reactive oxygen species (ROS)-NOD-like receptor family pyrin domain-containing 3 (NLRP3) signaling pathway, which decreases the insulin signaling and results in T2DM (47).

Previous studies have confirmed that the polarization of adipose macrophages from anti-inflammatory (M2) macrophages into proinflammatory (M1) macrophages is pivotal for obesity-induced insulin resistance (48–50). However, it is now generally agreed that the proliferation and activation of NK cells in VAT in the context of obesity also play important roles in insulin resistance and T2DM (14, 51). As discussed above, NK cells in obesity are involved in a feedback loop through an interaction with ATMs, triggering and amplifying the secretion of inflammatory cytokines, such as IL-6 and TNF-α, by these macrophages (9). Cytokines influence IκB kinase-β (IKKβ) and JUN N-terminal kinase (JNK) in adjacent cells, which can phosphorylate insulin receptor (IR)/insulin receptor substrate (IRS), hindering phosphatidylinositol 3-kinase (PI3K) and the protein kinase B (PKB)/Akt pathway downstream (52–54). These changes reduce the intake of glucose into cells by blocking the translocation of the glucose transporter GLUT4 to the cell membrane and promote free fatty acid production by inhibiting hormone-sensitive lipase (47). Furthermore, activated IKKβ also promotes NF-κB signaling, which is a well-known proinflammatory pathway that transcriptionally induces the production of more IL-6 and TNF-α for secretion by adipocytes (55, 56). Ultimately, the extensive secretion of cytokines and release of free fatty acids into the circulation from adipose tissue in an endocrine manner results in insulin resistance in other tissues, such as the muscles and liver, leading to an elevated blood glucose level (57).

Therefore, several studies have revealed a protective effect limiting insulin resistance and T2DM when NK cells are depleted in the context of obesity (26, 58). For example, Lee, B. C et al. blocked murine NK cells *in vivo* with a neutralizing antibody, which obviously improved obesity-induced insulin resistance and glucose tolerance (26). The same results were also observed in E4bp4^+/-^ and NKp46^Cre^Stopcodon^fl/fl^-huDTR mice, which were genetically depleted of NK cells (26, 58). ATMs may be the key point during this process because their infiltration rather than that of T cells into adipose tissue significantly decreases when NK cells are depleted (26, 52, 53, 58).

## NK Cells in NAFLD

Although NK cells account for only 2–18% of the lymphocytes in human peripheral blood, they compose up to 30% of the lymphocytes in the liver, consisting of half CD56^bright^ and half CD56^dim^ cells with unique phenotypes and functions (59–61). Accompanied by obesity, NAFLD is the most common chronic liver disease worldwide and can progress from simple steatosis to nonalcoholic steatohepatitis (NASH), which is one of the major complications of NAFLD (62). Consistent with other metabolic diseases, inflammation is a major contributor to NAFLD and can be induced by toxicity caused by the influx of lipids, responses to hepatocyte death and bacterial products due to increased intestinal permeability in the context of obesity (11). It is clear that activated immune cells are vital components in the inflammatory microenvironment, and many studies have revealed that NK cells also participate in this process (11, 61, 63). In NAFLD, the recruitment and activation of NK cells by several NK cell-activating cytokines and ligands can be observed in the inflamed liver. For example, the IL-15-mediated accumulation of lipids and expression of the chemokines MCP-1, CCL5 and CXCL10 in NAFLD (64) can regulate NK cell and/or mononuclear cell recruitment upon inflammation. In addition, the gene expression of IL-18 is also upregulated in the NAFLD liver, along with the levels of IFN-γ and TNF-α. Although the source of the cytokines is not clear, the activated NK cells may be one of the major sources (65, 66).

However, in some studies, NK cells seem to play a protective role in the development of fibrosis caused by different etiologies through killing hepatic stellate cell-derived myofibroblasts or stressed hepatocytes with the engagement of NKG2D, NKp46, NKp30, and/or TNF-related apoptosis-inducing ligand (TRAIL), as well as the release of IFN-γ and IL-22 (61). Under NAFLD conditions, NK cells seem to be beneficial in obese livers. This beneficial effect is likely achieved at the cost of reduced cytotoxicity, as NK cell cytotoxicity-deficient mice suffer from less severe NAFLD, which is partially mediated by the higher TGF-β level in obese liver tissue (67).

Stiglund N and his colleagues reported retained NK cell functions and increased activating receptor expression in obese NAFLD patients (63). They found that in NAFLD patients, circulating NK cells upregulate expression of the activating receptor NKG2D but there was no difference in NK cell functions or phenotypes between these patients and healthy individuals. They attributed these phenomena to the hypothesis that alterations in NK cell function or phenotype became evident only in patients with morbid obesity, not those with less severe obesity (63). Accordingly, the number of hepatic NK cells, their expression of NKG2D and the levels of the corresponding ligand of NKG2D, Major histocompatibility complex class I chain-related (MIC) A/B transcripts, are all increased in NASH patients with morbid obesity, being higher than those in patients with a nonalcoholic fatty liver (68). In a clinical case-control study, Iorio et al. showed that children with obesity-related liver disease had significantly higher peripheral NK cell levels than isolated obese children without liver diseases (69). Indeed, it is widely accepted that immune cell responses usually vary at different stages of pathology (70). This evidence indicates that the roles of NK cells become increasingly important during disease progression, and this undoubtedly increases the significance of studies in this field.

## NK Cells in Atherosclerosis

Atherosclerotic lesions contain NK cells even if the person is not obese. The presence of NK cells in both human and mouse atherosclerotic lesions, especially at the necrotic cores in advanced atherosclerotic plaques, was confirmed more than ten years ago (71, 72). NK cells are believed to compose only approximately 0.1–0.5% of the total lymphocytes present in atherosclerotic plaques and are frequently found in regions near necrotic cores deep within plaques and in the shoulder regions (71, 73). NK cells are recruited to the lesions *via* a possible chemokine-chemokine receptor gradient, such as MCP-1 and fractalkine (CX3CL1) gradients (74). Initial studies using the beige-mutant (total NK cell defective mice) and Ly49A-transgenic mouse models (functional NK cell defective mice) to deplete NK cells showed decreased atherosclerosis (72, 75). In addition, by depleting NK cells by anti–GM1 antibodies or transferring NK cells in atherosclerosis-prone ApoE-deficient mice, NK cells were demonstrated to play a protective role in the development of atherosclerosis, possibly *via* secretion of the cytotoxic molecules perforin and granzyme B (76). However, these strategies could not exclude the role of other lymphocytes in the development of atherosclerosis (76). For example, beige mutant mice also include defects in neutrophils, macrophages, or smooth muscle cells (77); in Ly49A transgenic mice, the role of natural killer T cells (NKT) and CD8 subsets, whose functions are influenced by Ly49A, were not considered (78, 79); GM1 is also expressed by myeloid cells, epithelial cells, and T cell subsets, and so on (80–83). A recent study, by using Ncr1^iCre/+^R26^lsl−DTA/+^ mice that specially deplete NK cells and Noé mice in which NK cells are hyperresponsive, demonstrated that NK cells showed no direct effect on the natural development of hypercholesterolemia-induced atherosclerosis. However, NK cells play a pro-atherogenic role when an additional systemic NK cell overactivation occurs, such as systemic inflammation challenge (84).

As a strong activator of NK cells, IL-15 has been indicated to be atherogenic (85). It was found to be expressed in human and murine atherosclerotic lesions, and blockade of IL-15 using oral vaccination in hypercholesterolemic diet-fed Ldlr^-/-^ mice induced a 75% reduction in lesion size (86–88). However, the question of whether, or to what extent, IL-15 has a downstream effect on NK cells in this context has not been addressed.

NK cells in the circulatory system or other inflamed tissues also influence the formation and progression of atherosclerosis. CD160 is an essential NK cell activating receptor that can induce cytolytic responses and high production of cytokines (89). The expression of CD160 on circulating NK cells in atherosclerosis patients is significantly increased, correlating with the augmented production of proinflammatory cytokines, including IFN-γ, TNF-α and IL-6 (90). As TNF-α has been reported to induce NK cell apoptosis, a higher level of TNF-α could be a possible explanation for the decreased NK cell numbers found in the peripheral blood (90, 91). The NKG2D/ligand interaction is likely to contribute to the reciprocal effects of NK cells and other cells in atherosclerosis, as it has been demonstrated that NKG2D ligand levels are upregulated in multiple organs in mouse models, particularly atherosclerotic aortae and inflamed livers, where accumulated abnormal metabolites and increased NK cell numbers could also be observed (92). Since several other types of immune cells, such as NKT cells and T lymphocytes, also express NKG2D and participate in the progression of atherosclerosis, the role of NK cells in this process needs to be further investigated (93).

The dysregulated NK cells functions observed in atherosclerosis may be attributable to abnormal lipid metabolism, which is also the major cause of plaque formation. Sterol regulatory element-binding proteins (SREBPs) are transcription factors that have central roles in lipid metabolism (94). A recent study revealed that SREBPs positively regulate NK cell growth, proliferation, granzyme B expression, and especially IFN-γ secretion in combination with glucose metabolism (95). Accordingly, it is possible that the increased lipid metabolism that occurs in obesity passively promotes the SREBP signaling pathway, resulting in the initiation of inflammation mediated by NK cells, which ultimately induces plaque formation in vascular walls. Additionally, the roles of NK cells in the initiation and development of atherosclerotic plaques have started to receive attention recently; however, the conclusions seem to be conflicting.

## NK Cells in Hypertension

Hypertension results from vascular dysfunction, and inflammation derived from perivascular adipose tissue seems to play a critical role in pathogenesis. Kossmann et al. first revealed that NK cells participate in vascular injury in hypertension (96). They found that angiotensin II induced NK cell recruitment into the aortic walls, which triggered vascular oxidative stress and other inflammatory cell recruitment and activation in situ through IFN-γ secretion. Some indirect evidence also suggested critical roles for NK cells in hypertension by introducing the NK cell gene complex derived from C57BL/6 mice into the genome of BALB/C mice. These chimeric BALB/C mice showed the same sensitivity to vascular injury as C57BL/6 mice in a hypertension model (97). The involvement of NK cells in hypertension may mainly depend on their interactions with monocytes/macrophages (98). In the vascular walls, NK cell-derived IFN-γ plays a major role in activating monocytes/macrophages and driving them toward inflammatory phenotypes, resulting in vascular dysfunction (99, 100). In turn, activated monocytes produce IL-12, which promotes generation of IFN-γ by NK cells. Interestingly, this feedback loop is highly dependent on the transcription factor T-bet, which is essential in NK cell secretion of IFN-γ and monocyte secretion of IL-12 (101). However, a key question regarding how the feedback loop is initiated when obesity occurs remains to be solved.

## Future Challenges and Prospects for Targeting NK Cells in Metabolic Diseases

Although some progress has been made in fully understanding the unique phenotype and functions of NK cells in obesity and its comorbidities, multiple key questions remain unanswered and need to be addressed. First, many other immune and nonimmune cells coexist in adipose tissue and constantly interact with each other to induce local and systemic inflammation and the development of obesity and its related metabolic diseases (70, 102, 103). Therefore, to what extent NK cells contribute to these pathological processes and whether drugs targeting certain points in signaling pathways are efficient enough to reverse disorders in these situations need to be explored. Second, NK cells are a heterogeneous population and express unique proteins depending on the resident tissue and pathology (104, 105). In addition, the number and activation status of NK cells change in the diverse stages of the development of specific diseases, especially NAFLD. Considering these facts, the studies that have attempted to accurately elucidate the phenotypes and functions of NK cells in different pathological stages and diseases are far from sufficient. Third, immuno-metabolism is a young but promising field focused on understanding the important roles of immunity in metabolic regulation and metabolism in immune regulation. Defects in the metabolism of NK cells can contribute to the generation of NK cell dysfunction, but the field of immunotherapeutic strategies based on this transformational metabolism is still in its infancy (106). In summary, all of these difficulties reflect the complexity of the regulatory network in obesity, which may be one potential reason accounting for the disappointing clinical trials of anti-inflammatory agents utilized for metabolic disease treatment. Therefore, to advance this field, the questions mentioned above need to be further investigated.

NK cells are considered to be heterogeneous, which can partly be attributed to the multiple KIR haplotype genotypes in different subtypes (107). There are two basic groups of KIR haplotypes, termed A and B. Haplotype A encodes predominantly inhibitory receptors, while haplotype B consists of more genes and mainly encodes activating KIRs (108). The identification of KIR haplotypes is receiving increasing attention, since certain haplotypes have been verified to be related to the diagnosis or prognosis of some diseases. For example, with regard to hematological diseases, haplotype A has been reported to independently predict a high risk of disease progression in myelodysplastic syndrome patients, and haplotype B is believed to be associated with poor inducible responses in pediatric acute lymphoblastic leukemia (105, 107). Using the PCR-SSP method, Chinniah R et al. revealed the existence of different KIR genotypes and KIR-HLA receptor combinations between T2DM patients and healthy controls (109). Considering that KIR diversity in diseases is of significance and that this field is still in its infancy, to fully understand its heterogeneity and convert these results into clinical applications, more research needs to be done.

Precise immunotherapies based on targeting NK cells and their signaling pathways have been explored for a long time, and the most studied field is cancer. In this condition, major therapeutic strategies include (1) adoptive transfer of NK cells into patients (2); mobilization of NK cells with antibodies, small-molecule drugs, cytokines and adjuvants; and (3) modulation of adjacent cells related to NK cells through ligands or soluble factors present in the microenvironment (110). As discussed above, the interactions between NKG2D, NKp46 and their ligands are involved in obesity-related metabolic diseases to various degrees. Addressing the specific ligands for NKG2D or NKp46 in obese individuals may provide promising references for the treatment of metabolic diseases.

The crosstalk between ATMs and NK cells is pivotal in the initiation of obesity-induced inflammation. Therefore, it may be possible to block the crosstalk by targeting macrophages because they are easier to be artificially modified with the help of various strategies, especially the drug-delivery system (111, 112), including nanoparticles, liposomes, glucan shell microparticles or oligopeptide complexes (113), which utilize the high phagocytic capability of macrophages. For example, considering the reported CD163 antibody-coated liposomes to target M2-like macrophages (114), it may be possible to design Rae-1 blocking-antibody-coated liposomes that contain macrophage apoptosis-induced agents. Thus, once injected *in vivo*, the liposomes will specifically recognize and bind to Rae-1 that is expressed on ATMs, promoting phagocytosis of the carried apoptosis-induced agents into macrophages. In this way, the Rae-1^+^ ATMs might be cleaned specifically, thus blocking their crosstalk with NKG2D on NK cells.

## Conclusion

Although NK cells were previously considered to be a less prominent immune component in obesity and its comorbidities, the roles played by NK cells in these diseases have attracted a great deal of attention recently. Understanding the connections among the altered functions of NK cells, chronic inflammation initiated and maintained by adipose tissue and the influences of these events on obesity and its related metabolic diseases will provide a new alternative strategy to solve these problems. Although some achievements have been made to date, there is still a long way to go to achieve translation from laboratory results to clinical application.

## Author Contributions

The work presented was performed in collaboration by all authors. YL and FW analyzed the data and wrote the manuscript. SI and LT discussed and revised the manuscript. YD and YC devised the concept and revised the paper. All authors contributed to the article and approved the submitted version.

## Funding

This study was supported by grants from the National Natural Science Foundation of China (No. 81922068 to YD; No. 81970383 to YC), and the National Key Research and Development Project (No. 2020YFA0113500 to YD).

## Conflict of Interest

The authors declare that the research was conducted in the absence of any commercial or financial relationships that could be construed as a potential conflict of interest.
